# Synthesis, crystal structures and Hirshfeld surface analysis of 1,4-dibenzyl-6-methyl-1,4-di­hydro­quinoxaline-2,3-dione

**DOI:** 10.1107/S2056989020009895

**Published:** 2020-07-24

**Authors:** Emine Berrin Cinar, Ayman Zouitini, Youssef Kandri Rodi, Younes Ouzidan, Jérôme Marrot, Damien Prim, Necmi Dege, Eiad Saif

**Affiliations:** a Department of Physics, Faculty of Arts and Sciences, Ondokuz Mayıs University, Samsun, 55200, Turkey; b Laboratoire de Chimie Organique Appliquée, Université Sidi Mohamed Ben Abdallah, Faculté des Sciences et Techniques, BP 2202, Fez, Morocco; c Laboratoire de Chimie Physique et Chimie Bio-organique, Faculté des Sciences et Techniques Mohammedia, Université Hassan II, Casablanca, BP 146, 28800, Mohammedia, Morocco; d Institut Lavoisier de Versailles, UVSQ, CNRS, Université Paris-Saclay, 78035 Versailles, France; e Department of Computer and Electronic Engineering Technology, Sana’a Community College, Sana’a, Yemen

**Keywords:** crystal structure, Hirshfeld surfaces, quinoxaline, hydrogen bonding

## Abstract

The title quinoxaline mol­ecule, C_23_H_20_N_2_O_2_, is not planar, the dihedral angle angle between the mean planes of the benzene rings being 72.54 (15)°. In the crystal, mol­ecules are connected into chains extending parallel to (10) by weak C—H⋯O hydrogen bonds. Weak C—H⋯π inter­actions link the chains, forming a three-dimensional network structure. Hirshfeld surface analysis revealed that the most important contributions for the crystal packing are from H⋯H (48.7%), H⋯C/C⋯H (32.0%), H⋯O/O⋯H (15.4%), C⋯C (1.9%), H⋯N/N⋯H (1.1%) contacts.

## Abstract 2

In the title quinoxaline mol­ecule, the dihedral angle angle between the benzene rings is 72.54 (15)°. In the crystal, mol­ecules are connected into chains extending parallel to (10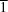) by weak C—H⋯O hydrogen bonds.

## Chemical context   

Given their importance in the pharmaceutical, chemical and industrial fields, the synthesis of quinoxaline and its derivatives has been a goal of chemists in recent years. Quinoxaline derivatives find use as anti­cancer (Noolvi *et al.*, 2011 ▸), anti­malarial (Guillon *et al.*, 2004 ▸), anti­fungal (Xu & Fan, 2011 ▸), anti­viral (Cai *et al.*, 2008 ▸) and anti-inflammatory (Yan *et al.*, 2007 ▸) agents. Some quinoxaline derivatives have also been reported to be corrosion inhibitors for steel in an acidic medium (Zouitini *et al.*, 2018 ▸, 2019 ▸; El Janati *et al.*, 2020 ▸). In this work, we report the synthesis and structure of the title compound obtained by the action of benzyl chloride on 6-methyl-1,4-di­hydro­quinoxaline-2,3-dione in the presence of potassium carbonate and a catalytic qu­antity of tetra-*n*-butyl­ammonium bromide. A Hirshfeld surface analysis was also performed.
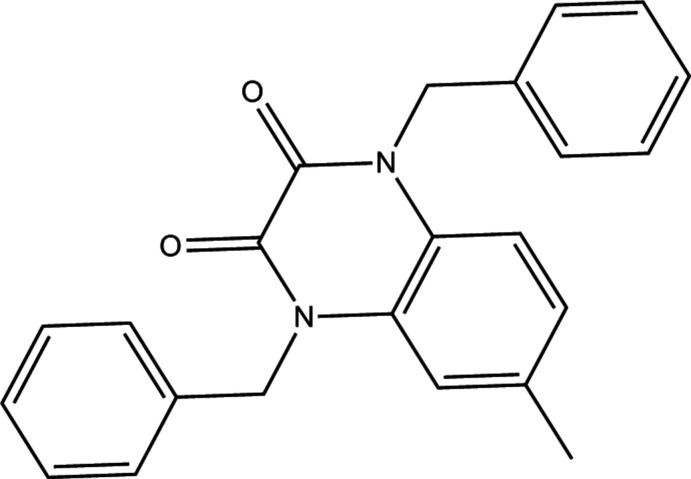


## Structural commentary   

**Figure 1:**
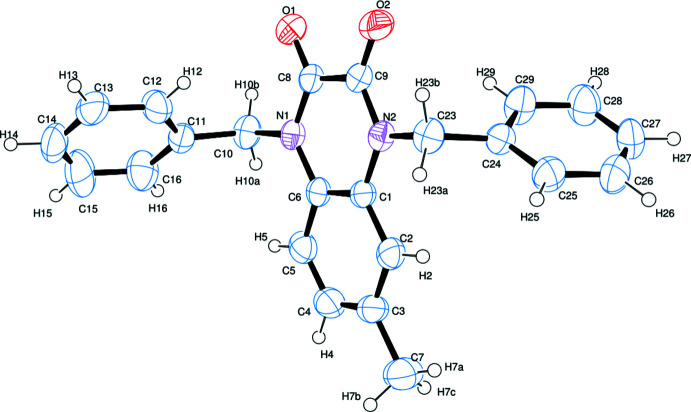
The mol­ecular structure of the title compound. Displacement ellipsoids are drawn at the 40% probability level.

An *ORTEPIII* (Burnett & Johnson, 1996 ▸) view of the mol­ecule is given in Fig. 1 ▸. The mol­ecule is not planar, the dihedral angle angle between the mean planes of the benzene rings (C11–C16 and C24–C29) being 72.54 (15)°. The mean planes of the C1/N2/C9/C8/N1/C6 and C11–C16 rings make an angle of 73.093 (13)° while the C1–C6 and C24–C29 rings make an angle of 79.01 (14)°. The C1/N2/C9/C8/N1/C6 and C1–C6 rings are nearly coplanar, subtending a dihedral angle of only 3.07 (11)°. The C8=O1 and C9=O2 bonds show double-bond character with bond lengths of 1.222 (3) and 1.217 (3) Å, respectively. The N1—C10 and N2—C23 bond lengths are 1.476 (3) and 1.464 (3) Å, respectively while the C11—C10—N1 bond angle is 113.57 (18)° and the N2—C23—C24 bond angle is 114.05 (18)°. The C9—N2—C23—C24 and C8—N1—C10—C11 torsion angles are −96.7 (2) and −93.4 (2)°, respectively.

## Supra­molecular features   

**Figure 2:**
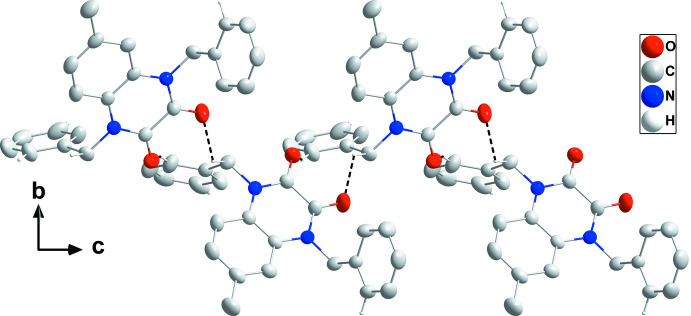
View of a portion of a chain along the *a*-axis direction with C—H⋯O hydrogen bonds depicted by dashed lines.

**Figure 3:**
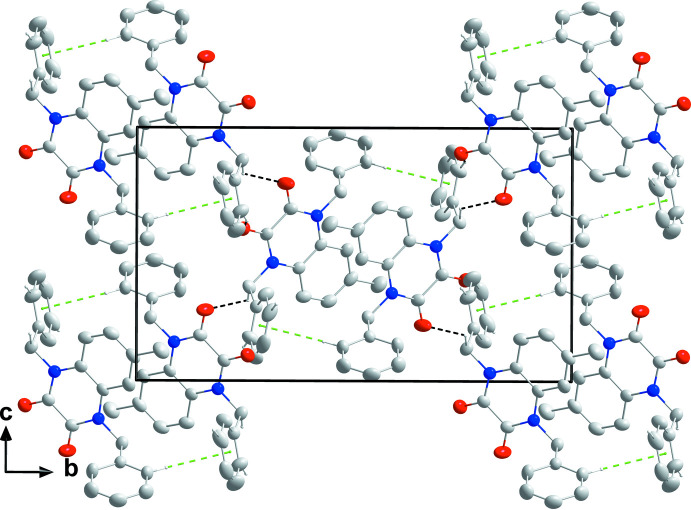
Packing viewed along the *a*-axis direction with C—H⋯O hydrogen bonds and C—H⋯π(ring) inter­actions depicted, respectively, by black and green dashed lines.

In the crystal, mol­ecules are connected by weak C16—H16⋯O1 and C10—H10*A*⋯O2 hydrogen bonds into chains extending parallel to (10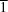) (Table 1 ▸ and Fig. 2 ▸). Weak C25—H25⋯*Cg*3 inter­actions (2.83 Å; *Cg*3 is the centroid of the C11–C16 ring at −*x* + 2, −*y*, −*z*) link the chains into a three-dimensional network structure (Table 1 ▸ and Fig. 3 ▸).

## Hirshfeld surface analysis   

**Figure 4:**
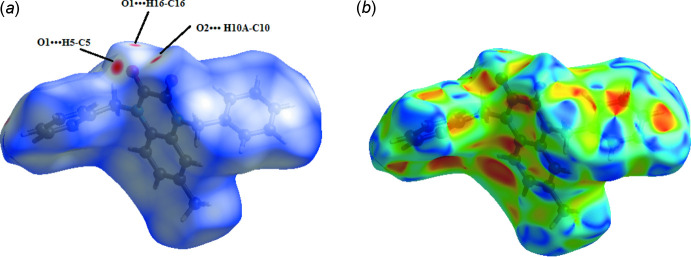
Hirshfeld surface mapper over (*a*) *d*_norm_ and (*b*) shape-index to visualize the inter­actions in the title compound.

**Figure 5:**
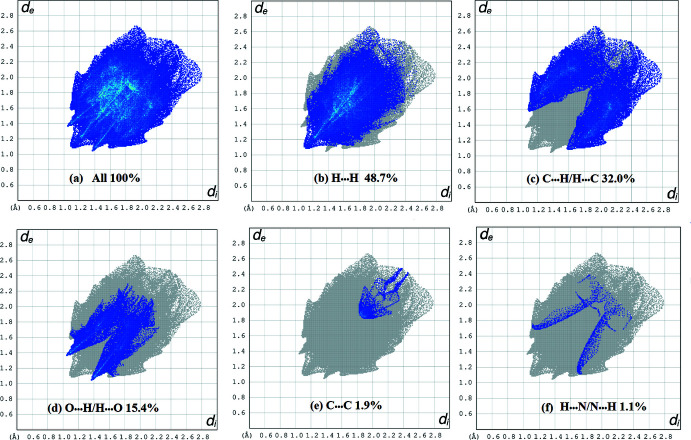
Fingerprint plot for all inter­actions and those delineated into the most important inter­actions.

The *CrystalExplorer17.5* (Turner *et al.*, 2017 ▸) program was used to analyse the inter­actions within the crystal. The donor–acceptor groups are visualized using a standard (high) surface resolution and *d_norm_* surfaces mapped over a fixed colour scale of −0.140 (red) to 1.358 (blue) a.u., as illustrated in Fig. 4 ▸. Red spots on the surface of the *d*_norm_ plot indicate inter­molecular contacts involving the hydrogen bonds. The red spots identified in Fig. 4 ▸(*a*) correspond to the inter­molecular C—H⋯O bonds. Regions close to the sum of the van der Waals radii are shown in white. Fig. 4 ▸(*b*) shows the shape-index surface, which can be used to detect the presence of π-stacking inter­actions. The absence of characteristic triangles indicates that no significant π–π inter­actions are present. Two-dimensional fingerprints were also generated in the range −1 to 1 Å (Fig. 5 ▸). As expected, H⋯H (48.7%) and H⋯C/C⋯H (32.0%) contacts dominate the inter­molecular inter­actions, but the O⋯H/H⋯O contacts are important directional inter­molecular inter­actions in the crystal. The C⋯C (1.9%) and H⋯N/N⋯H (1.1%) contribute minimally to the overall crystal packing.

## Database survey   

A search of the Cambridge Structural Database (CSD, version 5.40, update August 2019; Groom *et al.*, 2016 ▸) using 1-benzyl-3,4-di­hydro­quinoxalin-2(1*H*)-one as the main skeleton revealed the presence of three structures similar to the title compound, but with different substituents. These are: 1,4,6-tribenzoyl-3-(4-bromo­benz­yl)-1,4-di­hydro­quinoxaline-2-one (LEQWIO; Abraham *et al.*, 2006 ▸), 1,4-dibenzoyl-6-tri­fluoro­methyl-3-(4-bromo­benz­yl)-1,4-di­hydro­quinoxaline-2-one (LEQWOU; Abraham *et al.*, 2006 ▸) and 1,4-dibenzyl-6-chloro-1,4-di­hydro­quinoxaline-2,3-dione (PAWFEB; El Janati *et al.*, 2017 ▸). In the latter study (PAWFEB) examining compounds having the same skeletal system as the 1,4-di­hydro­quinoxaline-2,3-dione structure in the title compound, the corrosion inhibition efficiency of 1,4-diallyl-6-chloro­quin­oxaline-2,3-(1*H*,4*H*)-dione and 1,4-diallyl-6-nitro­quinoxaline-2,3-(1*H*,4*H*)-dione on mild steel (MS) in 1.0 *M* HCl solution was investigated.

## Synthesis and crystallization   

To a solution of 6-methyl-1,4-di­hydro­quinoxaline-2,3-dione (0.3 g, 1.73 mmol) in DMF (15 mL), were added potassium carbonate (0.47 g, 3.61 mmol) and tetra-*n*butyl­ammonium bromide (0.07g, 0.23 mmol). After stirring for 10 min, 0.5 mL (4.32 mmol) of benzyl chloride was added and the mixture was stirred at room temperature for 6 h. After filtration of the salts, the DMF was evaporated under reduced pressure and the residue obtained was dissolved in di­chloro­methane. The organic phase was then dried over Na_2_SO_4_ and concentrated. The mixture obtained was chromatographed on a silica gel column [eluent: hexa­ne/ethyl­acetate (2/1)]. The crude product was recrystallized from ethanol as yellow crystals suitable for X-ray analysis (m.p. 493.5 K).

## Refinement   

Crystal data, data collection and structure refinement details are summarized in Table 2 ▸. Hydrogen atoms treated as riding: C—H = 0.97 Å and *U*_iso_(H) = 1.5*U*_eq_(C) for methyl, C—H = 0.96 Å and *U*_iso_(H) = 1.2*U*_eq_(C) for methyl­ene, C—H = 0.93 Å and *U*_iso_(H) = 1.2*U*_eq_(C) for aromatic and C—H = 0.98 Å and *U*_iso_(H) = 1.2*U*_eq_(C) for methine H atoms.

## Supplementary Material

Crystal structure: contains datablock(s) I. DOI: 10.1107/S2056989020009895/mw2165sup1.cif

Structure factors: contains datablock(s) I. DOI: 10.1107/S2056989020009895/mw2165Isup3.hkl

Supporting information file. DOI: 10.1107/S2056989020009895/mw2165Isup3.cml

CCDC reference: 1936664

Additional supporting information:  crystallographic information; 3D view; checkCIF report

## Figures and Tables

**Table 1:** Hydrogen-bond geometry (Å, °) *Cg*3 is the centroid of the C11–C16 benzene ring.

*D*—H⋯*A*	*D*—H	H⋯*A*	*D*⋯*A*	*D*—H⋯*A*
C10—H10*A*⋯O2^i^	0.97	2.52	3.251 (3)	132
C16—H16⋯O1^i^	0.93	2.55	3.3984 (4)	152
C25—H25⋯*Cg*3^ii^	0.93	2.83	3.683 (3)	153

Symmetry codes: (i) 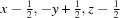; (ii) 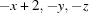.

**Table 2:** Experimental details

Crystal data	
Chemical formula	C_23_H_20_N_2_O_2_
*M*_r_	356.41
Crystal system, space group	Monoclinic, *P*2_1_/*n*
Temperature (K)	296
*a*, *b*, *c* (Å)	9.0844 (12), 18.7227 (18), 11.2708 (14)
β (°)	104.848 (4)
*V* (Å^3^)	1853.0 (4)
*Z*	4
Radiation type	Mo *K*α
μ (mm^−1^)	0.08
Crystal size (mm)	0.30 × 0.16 × 0.06

Data collection	
Diffractometer	Bruker APEXII CCD
Absorption correction	Multi-scan (*SADABS*; Bruker, 2016 ▸)
*T*_min_, *T*_max_	0.677, 0.746
No. of measured, independent and observed [*I* > 2σ(*I*)] reflections	22287, 4253, 2487
*R*_int_	0.054
(sin θ/λ)_max_ (Å^−1^)	0.649

Refinement	
*R*[*F* ^2^ > 2σ(*F* ^2^)], *wR*(*F* ^2^), *S*	0.060, 0.188, 1.04
No. of reflections	4253
No. of parameters	244
No. of restraints	1
H-atom treatment	H-atom parameters constrained
Δρ_max_, Δρ_min_ (e Å^−3^)	0.38, −0.25

Computer programs: *APEX3* and *SAINT* (Bruker, 2016 ▸), *SHELXT2018/3* (Sheldrick, 2015*a* ▸), *SHELXL2018/3* (Sheldrick, 2015*b* ▸), *OLEX2* (Dolomanov *et al.*, 2009 ▸), *Mercury* (Macrae *et al.*, 2020 ▸), *WinGX* (Farrugia, 2012 ▸), *PLATON* (Spek, 2020 ▸) and *publCIF* (Westrip, 2010 ▸).

## supplementary crystallographic information

### Crystal data

**Table:**

C_23_H_20_N_2_O_2_	*F*(000) = 752
*M**_r_* = 356.41	*D*_x_ = 1.278 Mg m^−^^3^
Monoclinic, *P*2_1_/*n*	Mo *K*α radiation, λ = 0.71073 Å
*a* = 9.0844 (12) Å	Cell parameters from 4963 reflections
*b* = 18.7227 (18) Å	θ = 2.6–24.6°
*c* = 11.2708 (14) Å	µ = 0.08 mm^−^^1^
β = 104.848 (4)°	*T* = 296 K
*V* = 1853.0 (4) Å^3^	Parallelepiped, yellow
*Z* = 4	0.30 × 0.16 × 0.06 mm

### Data collection

**Table:**

Bruker APEXII CCD diffractometer	2487 reflections with *I* > 2σ(*I*)
Radiation source: fine-focus sealed tube	*R*_int_ = 0.054
φ and ω scans	θ_max_ = 27.5°, θ_min_ = 2.2°
Absorption correction: multi-scan (SADABS; Bruker, 2016)	*h* = −10→11
*T*_min_ = 0.677, *T*_max_ = 0.746	*k* = −17→24
22287 measured reflections	*l* = −14→14
4253 independent reflections	

### Refinement

**Table:**

Refinement on *F*^2^	Primary atom site location: structure-invariant direct methods
Least-squares matrix: full	Secondary atom site location: difference Fourier map
*R*[*F*^2^ > 2σ(*F*^2^)] = 0.060	Hydrogen site location: inferred from neighbouring sites
*wR*(*F*^2^) = 0.188	H-atom parameters constrained
*S* = 1.04	*w* = 1/[σ^2^(*F*_o_^2^) + (0.0761*P*)^2^ + 0.7497*P*] where *P* = (*F*_o_^2^ + 2*F*_c_^2^)/3
4253 reflections	(Δ/σ)_max_ < 0.001
244 parameters	Δρ_max_ = 0.38 e Å^−^^3^
1 restraint	Δρ_min_ = −0.24 e Å^−^^3^

### Special details

**Table:**

Geometry. All esds (except the esd in the dihedral angle between two l.s. planes) are estimated using the full covariance matrix. The cell esds are taken into account individually in the estimation of esds in distances, angles and torsion angles; correlations between esds in cell parameters are only used when they are defined by crystal symmetry. An approximate (isotropic) treatment of cell esds is used for estimating esds involving l.s. planes.

### Fractional atomic coordinates and isotropic or equivalent isotropic displacement parameters (Å^2^)

**Table:**

	*x*	*y*	*z*	*U*_iso_*/*U*_eq_	
O1	0.6364 (2)	0.24430 (9)	0.60030 (16)	0.0668 (5)	
O2	0.6604 (2)	0.34222 (11)	0.77924 (17)	0.0787 (6)	
C1	0.3801 (2)	0.42320 (11)	0.5380 (2)	0.0453 (5)	
C2	0.2840 (3)	0.48294 (13)	0.5167 (2)	0.0565 (6)	
H2	0.290850	0.516663	0.578499	0.068*	
C3	0.1779 (3)	0.49239 (14)	0.4035 (3)	0.0606 (7)	
C4	0.1685 (3)	0.44202 (16)	0.3149 (3)	0.0665 (7)	
H4	0.096780	0.447513	0.240048	0.080*	
C5	0.2611 (3)	0.38383 (14)	0.3329 (2)	0.0605 (7)	
H5	0.253041	0.350819	0.269920	0.073*	
C6	0.3690 (2)	0.37294 (12)	0.4457 (2)	0.0463 (5)	
C7	0.0744 (4)	0.55686 (17)	0.3816 (3)	0.0857 (9)	
H7A	0.097017	0.586438	0.453541	0.129*	
H7B	0.090205	0.583627	0.313222	0.129*	
H7C	−0.029766	0.541485	0.364004	0.129*	
N1	0.4634 (2)	0.31227 (10)	0.46724 (17)	0.0479 (5)	
C8	0.5589 (3)	0.29843 (12)	0.5791 (2)	0.0502 (6)	
C9	0.5729 (3)	0.35335 (13)	0.6795 (2)	0.0530 (6)	
N2	0.4870 (2)	0.41352 (10)	0.65252 (17)	0.0477 (5)	
C10	0.4593 (3)	0.25871 (12)	0.3703 (2)	0.0564 (6)	
H10A	0.356426	0.255943	0.318086	0.068*	
H10B	0.484757	0.212320	0.408395	0.068*	
C11	0.5669 (3)	0.27494 (12)	0.2918 (2)	0.0520 (6)	
C12	0.7141 (3)	0.29739 (15)	0.3413 (3)	0.0682 (7)	
H12	0.746539	0.306858	0.424992	0.082*	
C13	0.8143 (4)	0.30607 (17)	0.2690 (3)	0.0839 (10)	
H13	0.913384	0.321355	0.303781	0.101*	
C14	0.7672 (5)	0.29203 (17)	0.1452 (4)	0.0907 (11)	
H14	0.834725	0.297218	0.096117	0.109*	
C15	0.6232 (6)	0.2708 (2)	0.0954 (3)	0.1045 (12)	
H15	0.590820	0.262012	0.011500	0.125*	
C16	0.5229 (4)	0.26191 (17)	0.1682 (3)	0.0820 (9)	
H16	0.423893	0.246823	0.132573	0.098*	
C23	0.5078 (3)	0.46790 (13)	0.7488 (2)	0.0561 (6)	
H23A	0.502294	0.514721	0.711069	0.067*	
H23B	0.608828	0.462678	0.803360	0.067*	
C24	0.3920 (3)	0.46424 (11)	0.8239 (2)	0.0486 (5)	
C25	0.3412 (3)	0.52590 (13)	0.8661 (2)	0.0641 (7)	
H25	0.373901	0.569841	0.844097	0.077*	
C26	0.2429 (4)	0.52371 (16)	0.9403 (3)	0.0785 (9)	
H26	0.208237	0.565956	0.967137	0.094*	
C27	0.1958 (4)	0.45942 (18)	0.9747 (3)	0.0799 (9)	
H27	0.129675	0.457886	1.025374	0.096*	
C28	0.2455 (4)	0.39791 (16)	0.9349 (3)	0.0777 (8)	
H28	0.214200	0.354182	0.959034	0.093*	
C29	0.3424 (3)	0.39995 (13)	0.8588 (3)	0.0666 (7)	
H29	0.374561	0.357497	0.830698	0.080*	

### Atomic displacement parameters (Å^2^)

**Table:**

	*U*^11^	*U*^22^	*U*^33^	*U*^12^	*U*^13^	*U*^23^
O1	0.0702 (12)	0.0659 (11)	0.0686 (12)	0.0218 (9)	0.0256 (9)	0.0050 (8)
O2	0.0710 (13)	0.1036 (15)	0.0554 (11)	0.0349 (11)	0.0050 (10)	−0.0041 (10)
C1	0.0359 (11)	0.0508 (12)	0.0539 (14)	0.0000 (9)	0.0197 (10)	0.0130 (10)
C2	0.0536 (14)	0.0563 (13)	0.0671 (16)	0.0013 (11)	0.0292 (13)	0.0090 (12)
C3	0.0433 (13)	0.0678 (15)	0.0736 (18)	0.0060 (11)	0.0204 (13)	0.0259 (14)
C4	0.0497 (15)	0.089 (2)	0.0604 (16)	0.0034 (14)	0.0131 (12)	0.0162 (15)
C5	0.0476 (14)	0.0820 (17)	0.0528 (15)	−0.0043 (13)	0.0149 (12)	0.0033 (13)
C6	0.0370 (11)	0.0550 (12)	0.0514 (13)	−0.0041 (10)	0.0197 (10)	0.0057 (10)
C7	0.071 (2)	0.090 (2)	0.094 (2)	0.0186 (17)	0.0178 (17)	0.0233 (18)
N1	0.0414 (10)	0.0542 (11)	0.0525 (12)	−0.0031 (8)	0.0200 (9)	0.0006 (9)
C8	0.0458 (13)	0.0563 (13)	0.0540 (14)	0.0056 (11)	0.0231 (11)	0.0057 (11)
C9	0.0449 (13)	0.0656 (15)	0.0518 (14)	0.0104 (11)	0.0184 (12)	0.0029 (11)
N2	0.0442 (10)	0.0510 (10)	0.0519 (11)	0.0002 (8)	0.0194 (9)	−0.0012 (8)
C10	0.0487 (14)	0.0588 (14)	0.0650 (16)	−0.0127 (11)	0.0204 (12)	−0.0112 (12)
C11	0.0514 (14)	0.0517 (12)	0.0570 (14)	0.0012 (11)	0.0213 (11)	−0.0011 (11)
C12	0.0526 (15)	0.0883 (19)	0.0666 (17)	−0.0084 (14)	0.0205 (13)	0.0175 (14)
C13	0.0586 (17)	0.087 (2)	0.117 (3)	0.0116 (15)	0.0420 (18)	0.0379 (19)
C14	0.116 (3)	0.0689 (18)	0.117 (3)	0.0319 (19)	0.084 (3)	0.0182 (18)
C15	0.143 (4)	0.109 (3)	0.082 (2)	−0.003 (3)	0.066 (3)	−0.024 (2)
C16	0.087 (2)	0.094 (2)	0.0682 (19)	−0.0083 (17)	0.0272 (16)	−0.0236 (16)
C23	0.0534 (14)	0.0531 (13)	0.0644 (15)	−0.0076 (11)	0.0198 (12)	−0.0054 (11)
C24	0.0478 (13)	0.0474 (12)	0.0513 (13)	−0.0012 (10)	0.0142 (10)	−0.0025 (10)
C25	0.0786 (19)	0.0498 (13)	0.0684 (17)	0.0069 (12)	0.0272 (15)	−0.0007 (12)
C26	0.099 (2)	0.0731 (18)	0.0750 (19)	0.0230 (17)	0.0426 (17)	−0.0042 (15)
C27	0.082 (2)	0.106 (2)	0.0638 (18)	0.0093 (18)	0.0411 (16)	0.0004 (17)
C28	0.090 (2)	0.0710 (18)	0.085 (2)	−0.0113 (16)	0.0457 (17)	0.0041 (15)
C29	0.0773 (18)	0.0492 (13)	0.0837 (19)	−0.0066 (12)	0.0396 (15)	−0.0059 (13)

### Geometric parameters (Å, º)

**Table:**

O1—C8	1.222 (3)	C16—H16	0.9300
O2—C9	1.217 (3)	C15—C14	1.345 (5)
C1—C6	1.388 (3)	C15—H15	0.9300
C1—C2	1.401 (3)	C14—C13	1.376 (5)
C1—N2	1.414 (3)	C14—H14	0.9300
C2—C3	1.400 (4)	C13—C12	1.378 (4)
C2—H2	0.9300	C13—H13	0.9300
C3—C4	1.360 (4)	C12—H12	0.9300
C3—C7	1.511 (4)	C23—C24	1.511 (3)
C4—C5	1.360 (4)	C23—H23A	0.9700
C4—H4	0.9300	C23—H23B	0.9700
C5—C6	1.407 (3)	C24—C25	1.373 (3)
C5—H5	0.9300	C24—C29	1.378 (3)
C6—N1	1.406 (3)	C29—C28	1.378 (4)
N1—C8	1.359 (3)	C29—H29	0.9300
N1—C10	1.476 (3)	C28—C27	1.355 (4)
C8—C9	1.510 (3)	C28—H28	0.9300
C9—N2	1.360 (3)	C27—C26	1.366 (4)
N2—C23	1.464 (3)	C27—H27	0.9300
C10—C11	1.507 (3)	C26—C25	1.371 (4)
C10—H10A	0.9700	C26—H26	0.9300
C10—H10B	0.9700	C25—H25	0.9300
C11—C16	1.370 (4)	C7—H7A	0.9600
C11—C12	1.376 (4)	C7—H7B	0.9600
C16—C15	1.384 (5)	C7—H7C	0.9600
			
C6—C1—C2	119.4 (2)	C14—C15—C16	120.4 (3)
C6—C1—N2	119.92 (19)	C14—C15—H15	119.8
C2—C1—N2	120.7 (2)	C16—C15—H15	119.8
C3—C2—C1	120.7 (2)	C15—C14—C13	119.7 (3)
C3—C2—H2	119.7	C15—C14—H14	120.1
C1—C2—H2	119.7	C13—C14—H14	120.1
C4—C3—C2	118.7 (2)	C14—C13—C12	119.8 (3)
C4—C3—C7	121.0 (3)	C14—C13—H13	120.1
C2—C3—C7	120.3 (3)	C12—C13—H13	120.1
C5—C4—C3	121.7 (3)	C11—C12—C13	121.1 (3)
C5—C4—H4	119.1	C11—C12—H12	119.4
C3—C4—H4	119.1	C13—C12—H12	119.4
C4—C5—C6	120.8 (3)	N2—C23—C24	114.05 (18)
C4—C5—H5	119.6	N2—C23—H23A	108.7
C6—C5—H5	119.6	C24—C23—H23A	108.7
C1—C6—N1	119.6 (2)	N2—C23—H23B	108.7
C1—C6—C5	118.6 (2)	C24—C23—H23B	108.7
N1—C6—C5	121.7 (2)	H23A—C23—H23B	107.6
C8—N1—C6	122.01 (19)	C25—C24—C29	118.2 (2)
C8—N1—C10	116.47 (19)	C25—C24—C23	120.0 (2)
C6—N1—C10	121.47 (19)	C29—C24—C23	121.7 (2)
O1—C8—N1	122.6 (2)	C28—C29—C24	120.7 (2)
O1—C8—C9	118.9 (2)	C28—C29—H29	119.7
N1—C8—C9	118.5 (2)	C24—C29—H29	119.7
O2—C9—N2	123.4 (2)	C27—C28—C29	120.2 (3)
O2—C9—C8	119.1 (2)	C27—C28—H28	119.9
N2—C9—C8	117.6 (2)	C29—C28—H28	119.9
C9—N2—C1	122.08 (19)	C28—C27—C26	120.0 (3)
C9—N2—C23	116.9 (2)	C28—C27—H27	120.0
C1—N2—C23	121.01 (19)	C26—C27—H27	120.0
N1—C10—C11	113.57 (18)	C27—C26—C25	119.9 (2)
N1—C10—H10A	108.9	C27—C26—H26	120.0
C11—C10—H10A	108.9	C25—C26—H26	120.0
N1—C10—H10B	108.9	C26—C25—C24	121.0 (2)
C11—C10—H10B	108.9	C26—C25—H25	119.5
H10A—C10—H10B	107.7	C24—C25—H25	119.5
C16—C11—C12	117.8 (2)	C3—C7—H7A	109.5
C16—C11—C10	119.8 (2)	C3—C7—H7B	109.5
C12—C11—C10	122.2 (2)	H7A—C7—H7B	109.5
C11—C16—C15	121.2 (3)	C3—C7—H7C	109.5
C11—C16—H16	119.4	H7A—C7—H7C	109.5
C15—C16—H16	119.4	H7B—C7—H7C	109.5
			
C6—C1—C2—C3	−0.2 (3)	C6—C1—N2—C9	−5.1 (3)
N2—C1—C2—C3	−179.82 (19)	C2—C1—N2—C9	174.47 (19)
C1—C2—C3—C4	0.8 (3)	C6—C1—N2—C23	176.09 (18)
C1—C2—C3—C7	179.8 (2)	C2—C1—N2—C23	−4.3 (3)
C2—C3—C4—C5	−1.3 (4)	C8—N1—C10—C11	−93.4 (2)
C7—C3—C4—C5	179.7 (2)	C6—N1—C10—C11	88.9 (2)
C3—C4—C5—C6	1.2 (4)	N1—C10—C11—C16	−141.3 (2)
C2—C1—C6—N1	−178.95 (18)	N1—C10—C11—C12	44.0 (3)
N2—C1—C6—N1	0.6 (3)	C12—C11—C16—C15	0.2 (4)
C2—C1—C6—C5	0.1 (3)	C10—C11—C16—C15	−174.7 (3)
N2—C1—C6—C5	179.71 (18)	C11—C16—C15—C14	0.5 (5)
C4—C5—C6—C1	−0.6 (3)	C16—C15—C14—C13	−1.0 (5)
C4—C5—C6—N1	178.5 (2)	C15—C14—C13—C12	0.8 (5)
C1—C6—N1—C8	4.6 (3)	C16—C11—C12—C13	−0.4 (4)
C5—C6—N1—C8	−174.4 (2)	C10—C11—C12—C13	174.4 (2)
C1—C6—N1—C10	−177.85 (18)	C14—C13—C12—C11	−0.1 (4)
C5—C6—N1—C10	3.1 (3)	C9—N2—C23—C24	−96.7 (2)
C6—N1—C8—O1	176.7 (2)	C1—N2—C23—C24	82.2 (2)
C10—N1—C8—O1	−1.0 (3)	N2—C23—C24—C25	−144.8 (2)
C6—N1—C8—C9	−5.3 (3)	N2—C23—C24—C29	39.5 (3)
C10—N1—C8—C9	177.02 (19)	C25—C24—C29—C28	−0.6 (4)
O1—C8—C9—O2	−0.4 (3)	C23—C24—C29—C28	175.1 (3)
N1—C8—C9—O2	−178.5 (2)	C24—C29—C28—C27	1.2 (5)
O1—C8—C9—N2	179.0 (2)	C29—C28—C27—C26	−0.6 (5)
N1—C8—C9—N2	0.9 (3)	C28—C27—C26—C25	−0.4 (5)
O2—C9—N2—C1	−176.4 (2)	C27—C26—C25—C24	1.0 (5)
C8—C9—N2—C1	4.2 (3)	C29—C24—C25—C26	−0.4 (4)
O2—C9—N2—C23	2.4 (3)	C23—C24—C25—C26	−176.2 (3)
C8—C9—N2—C23	−176.91 (19)		

### Hydrogen-bond geometry (Å, º)

*Cg*3 is the centroid of the C11–C16 benzene ring.

**Table:**

*D*—H···*A*	*D*—H	H···*A*	*D*···*A*	*D*—H···*A*
C10—H10*A*···O2^i^	0.97	2.52	3.251 (3)	132
C16—H16···O1^i^	0.93	2.55	3.3984 (4)	152
C25—H25···*Cg*3^ii^	0.93	2.83	3.683 (3)	153

Symmetry codes: (i) *x*−1/2, −*y*+1/2, *z*−1/2; (ii) −*x*+2, −*y*, −*z*.
